# [Expression of Concern] Knockdown of Bmi1 inhibits the stemness properties and tumorigenicity of human bladder cancer stem cell-like side population cells

**DOI:** 10.3892/or.2025.9006

**Published:** 2025-10-14

**Authors:** Dingjun Zhu, Xuesi Wan, Hai Huang, Xu Chen, Wu Liang, Fengjin Zhao, Tianxin Lin, Jinli Han, Wenlian Xie

Oncol Rep 31: 727–736, 2014; DOI: 10.3892/or.2013.2919

Following the publication of this paper, a concerned reader drew to the Editor's attention that the positioning and proportions of the first and third mice (from the left) in Fig. 7A were very similar, raising a possible concern that the same mouse had been used to show the experiments in these cases.

The authors were contacted by the Editorial Office to offer an explanation for this potential anomaly in the presentation of the data in this paper; however, up to this time, no response from them has been forthcoming. Owing to the fact that the Editorial Office has been made aware of potential issues surrounding the scientific integrity of this paper, we are issuing an Expression of Concern to notify readers of this potential problem while the Editorial Office continues to investigate this matter further.

